# Supplementary material for: Perpendicular electric field drives Chern transitions and layer polarization changes in Hofstadter bands
Source: Nat Commun. 2022 Dec 16;13:7781. doi: 10.1038/s41467-022-35421-z (PMC9758152; doi:10.1038/s41467-022-35421-z)
Supplement: Supplementary file 1 — Supplementary Information [file 41467_2022_35421_MOESM1_ESM.pdf]

SUPPLEMENTARY INFORMATION

Perpendicular electric field drives Chern transitions and layer polarization changes in Hofstadter bands

Pratap Chandra Adak[1,*], Subhajit Sinha[1], Debasmita Giri[2], Dibya Kanti Mukherjee[3,4,5], Chandan[1], L. D. Varma Sangani[1], Surat Layek[1], Ayshi Mukherjee[1], Kenji Watanabe[6], Takashi Taniguchi[7], H.A. Fertig[3,4], Arijit Kundu[2,*], and Mandar M. Deshmukh[1,*]

[1]*Department of Condensed Matter Physics and Materials Science, Tata Institute of Fundamental Research, Homi Bhabha Road, Mumbai 400005, India.*

[2]*Department of Physics, Indian Institute of Technology, Kanpur 208016, India.*

[3]*Department of Physics, Indiana University, Bloomington, IN 47405.*

[4]*Quantum Science and Engineering Center, Indiana University, Bloomington, IN, 47408.*

[5]*Laboratoire de Physique des Solides, Univ. Paris-Sud, Université Paris Saclay, CNRS, UMR 8502, F-91405 Orsay Cedex, France.*

[6]*Research Center for Functional Materials, National Institute for Materials Science, 1-1 Namiki, Tsukuba 305-0044, Japan.*

[7]*International Center for Materials Nanoarchitectonics, National Institute for Materials Science, 1-1 Namiki, Tsukuba 305-0044, Japan.*

[*]pratapchandraadak@gmail.com, kundua@iitk.ac.in, deshmukh@tifr.res.in

Supplementary Note 1: Calculation of TDBG band structure at zero magnetic field

The continuum Hamiltonian[1–7] for twisted $AB-AB$ double bilayer graphene at small twist angle θ is written in a 8×8 matrix in the basis of the sublattices of four graphene layers (A_1, B_1, A_2, B_2, A_3, B_3, A_4, B_4) as,

$$H^{\xi}_{AB-AB} = \begin{pmatrix} H^{\xi}_1(\mathbf{k}_1) & g^{\dagger}(\mathbf{k}_1) & 0 & 0 \\ g(\mathbf{k}_1) & \tilde{H}^{\xi}_1(\mathbf{k}_1) & U & 0 \\ 0 & U^{\dagger} & \tilde{H}^{\xi}_2(\mathbf{k}_2) & g(\mathbf{k}_2) \\ 0 & 0 & g^{\dagger}(\mathbf{k}_1) & H^{\xi}_2(\mathbf{k}_2) \end{pmatrix} + \begin{pmatrix} \frac{3V}{2}I_2 & & & \\ & \frac{V}{2}I_2 & & \\ & & -\frac{V}{2}I_2 & \\ & & & -\frac{3V}{2}I_2 \end{pmatrix} \tag{1}$$

where

$$H^{\xi}_l(\mathbf{k}) = \begin{pmatrix} 0 & -\hbar v_F k^{\xi,l}_{-} \\ -\hbar v_F k^{\xi,l}_{+} & \Delta \end{pmatrix} \qquad \tilde{H}^{\xi}_l(\mathbf{k}) = \begin{pmatrix} \Delta & -\hbar v_F k^{\xi,l}_{-} \\ -\hbar v_F k^{\xi,l}_{+} & 0 \end{pmatrix} \tag{2}$$

are the monolayer graphene Hamiltonians and

$$g(\mathbf{k}) = \begin{pmatrix} \hbar v_4 k^{\xi,l}_{+} & \gamma_1 \\ \hbar v_3 k^{\xi,l}_{-} & \hbar v_4 k^{\xi,l}_{+} \end{pmatrix} \tag{3}$$

is the intra-bilayer coupling Hamiltonian. Here, $k^{\xi,l}_{\pm} = e^{i\eta^{\xi l}}(\xi k_x \pm i k_y)$ with $\eta^{1/2} = \mp\theta/2$. $\xi = \pm 1$ for $\mathbf{K}$ and $\mathbf{K}'$ valley respectively. v_3, v_4 are the velocities capturing the effect of the trigonal warping and electron-hole asymmetry respectively. Δ is the on-site potential felt by the vertically aligned lattice sites in the AB stacked bilayers and γ_1 is the coupling between the same sites. The tunneling matrix incorporating the twist between layers 2 and 3 is given by

$$U = \begin{pmatrix} t_0\alpha(\mathbf{r}) & t_1\beta(\mathbf{r}) \\ t_1\gamma(\mathbf{r}) & t_0\alpha(\mathbf{r}) \end{pmatrix}, \tag{4}$$

with $\alpha(\mathbf{r}) = \sum_{n=0}^{2} \exp(-i\xi\mathbf{G}_n.\mathbf{r})$, $\beta(\mathbf{r}) = \sum_{n=0}^{2} (w^n)^\xi \exp(-i\xi\mathbf{G}_n.\mathbf{r})$, $\gamma(\mathbf{r}) = \sum_{n=0}^{2} (w^{*n})^\xi \exp(-i\xi\mathbf{G}_n.\mathbf{r})$ and $w = \exp(i2\pi/3)$. $\mathbf{G}_n$ are the reciprocal lattice vectors of the moiré lattice, given by $\mathbf{G}_0 = 0$, $\mathbf{G}_1 = k_\theta(-\sqrt{3}/2, 3/2)$ and $\mathbf{G}_2 = k_\theta(\sqrt{3}/2, 3/2)$, and $k_\theta = |\mathbf{K}_2 - \mathbf{K}_3|$, where $\mathbf{K}_2$ and $\mathbf{K}_3$ denote the locations of Dirac points of layers 2 and 3 respectively with respect to a common origin. V is the applied electrostatic potential and I_2 is the 2×2 unit matrix. We use $t_0 = 0.05$ eV, $t_1 = 0.085$ eV, $\gamma_1 = 0.4$ eV, $\Delta = 0.05$ eV, $v_3 = 1.036 \times 10^5$ m/s, $v_4 = 0.143 \times 10^5$ m/s.

Supplementary Note 2: Calculation of Hofstadter energy spectra in TDBG

In presence of a perpendicular magnetic field $\mathbf{B}$=$B\hat{z}$, the wave function[8,9] of monolayer graphene at $\mathbf{K}$ and $\mathbf{K}'$ valley becomes,

$$\Psi_{n,\mathbf{K}} = c_n e^{-i\mathbf{K}_l.\mathbf{r}} e^{-iXy/l_B^2} \frac{1}{\sqrt{L_y}} \begin{pmatrix} -i \; sgn(n) \; \phi_{|n|-1}(x - X) \\ \phi_{|n|}(x - X) \end{pmatrix} \tag{5}$$

$$\Psi_{n,\mathbf{K}'} = c_n e^{-i\mathbf{K}'_l.\mathbf{r}} e^{-iXy/l_B^2} \frac{1}{\sqrt{L_y}} \begin{pmatrix} \phi_{|n|}(x - X) \\ -i \; sgn(n) \; \phi_{|n|-1}(x - X) \end{pmatrix} \tag{6}$$

and the corresponding landau level energy is, $\varepsilon_n = \frac{\hbar v_F}{l} sgn(n)\sqrt{2|n|}$.

Here $\phi_{|n|}(x - X) = (2^n n! \sqrt{\pi}\; l_B)^{-1/2} e^{-\frac{(x-X)^2}{2l_B^2}} H_n(\frac{x-X}{l_B})$, $H_n(\cdot)$ being the Hermite polynomial of order n. $X = -k_y l_B^2$ is the guiding centre coordinate, $l_B = (\frac{\Phi_0}{2\pi B})^{1/2}$ is the magnetic length scale. The normalization coefficient is $c_n = 1$ for $n = 0$ and $c_n = \frac{1}{\sqrt{2}}$ for $n \neq 0$. $\mathbf{K}_l$ are the moire Dirac points associated with each layer.

The diagonal elements of the matrix Hamiltonian are,

$$\langle n, X, \nu \mid H^d \mid n', X', \nu' \rangle = \varepsilon_n \delta_{n,n'} \delta_{X,X'} \delta_{\nu,\nu'} \tag{7}$$

where $|n, X, \nu\rangle$ denotes the n-th Landau level of the ν layer with its guiding center localized around X.

The intra-bilayer matrix elements (for $\mathbf{K}$ valley) are

$$\langle n, X, \nu \mid g(\mathbf{k})^\dagger \mid n', X', \nu' \rangle = c_n c_{n'} \Bigg[- i \ \mathrm{sgn}(nn') \frac{\sqrt{2}}{l_B} \hbar v_4 \sqrt{|n'| - 1} \ \delta_{|n|,|n'|-1} \ - \mathrm{sgn}(n) \frac{\sqrt{2}}{l_B} \hbar v_3 \sqrt{|n'| + 1} \ \delta_{|n|,|n'|+2}$$
$$- i \ \ \mathrm{sgn}(n') \gamma \delta_{|n|,|n'|-1} - i \frac{\sqrt{2}}{l_B} \hbar v_4 \sqrt{|n'|} \delta_{|n|,|n'|-1} \Bigg] \delta_{X,X'} \delta_{\nu,1} \delta_{\nu',2}. \tag{8}$$

The inter-bilayer matrix elements (for $\mathbf{K}$ valley) are found to be,

$$\langle n, X, \nu \mid U \mid n', X', \nu' \rangle = \Bigg[\delta_{X,X'-k_\theta l_B^2} \ A_{n,n'} \ e^{-iG'_{0,x}(X+X')/2} + \delta_{X,X'+k_\theta l_B^2/2} \ B_{n,n'} \ e^{-iG'_{1,x}(X+X')/2}$$
$$+ \delta_{X,X'+k_\theta l_B^2/2} \ D_{n,n'} \ e^{-iG'_{2,x}(X+X')/2} \Bigg] \delta_{\nu,2} \ \delta_{\nu',3} \tag{9}$$

where $\mathbf{G}'_i = \mathbf{G}_i - k_\theta \hat{y}$.

The functions,

$$A_{n,n'} = c_n c_{n'} \Bigg[t_0 \ \mathrm{sgn}(nn') f_{|n|-1,|n'|-1}(\mathbf{G}'_0) + t_0 f_{|n|,|n'|}(\mathbf{G}'_0) + i t_1 \ \mathrm{sgn}(n) f_{|n|-1,|n'|}(\mathbf{G}'_0)$$
$$- i t_1 \ \mathrm{sgn}(n') f_{|n|,|n'|-1}(\mathbf{G}'_0) \Bigg], \tag{10}$$

$$B_{n,n'} = c_n c_{n'} \Bigg[t_0 \ \mathrm{sgn}(nn') f_{|n|-1,|n'|-1}(\mathbf{G}'_1) + t_0 f_{|n|,|n'|}(\mathbf{G}'_1) + i t_1 w \ \mathrm{sgn}(n) f_{|n|-1,|n'|}(\mathbf{G}'_1)$$
$$- i t_1 w^* \ \mathrm{sgn}(n') f_{|n|,|n'|-1}(\mathbf{G}'_1) \Bigg] \tag{11}$$

$$D_{n,n'} = c_n c_{n'} \Bigg[t_0 \ \mathrm{sgn}(nn') f_{|n|-1,|n'|-1}(\mathbf{G}'_2) + t_0 f_{|n|,|n'|}(\mathbf{G}'_2) + i \ \mathrm{sgn}(n) \ t_1 w^* f_{|n|-1,|n'|}(\mathbf{G}'_2)$$
$$- i t_1 \ \mathrm{sgn}(n') w f_{|n|,|n'|-1}(\mathbf{G}'_2) \Bigg] \tag{12}$$

involve overlap between quantum states with different guiding centers and,

$$f_{n,n'}(\mathbf{G}) = \sqrt{\frac{m!}{M!}} \left(\frac{-G_x - iG_y}{|\mathbf{G}|}\right)^{n'-n} \left(i|\mathbf{G}|l_B/\sqrt{2}\right)^{|n-n'|} e^{-|\mathbf{G}|^2 l_B^2/4} L_m^{|n-n'|}(|\mathbf{G}|^2 l_B^2/2) \tag{13}$$

with $L_m^a(x)$ is an associated Laguerre polynomial and $m = \min(n, n')$, $M = \max(n, n')$.

It can be seen that the inter-bilayer Hamiltonian U couples quantum states with different guiding centers separated by $k_\theta l_B^2/2$. The information about the relative twist angle between the two bilayers thus gets encoded in the coupling between different guiding centers.

The full matrix Hamiltonian[9–11] for the $\mathbf{K}$ valley can be written as,

$$H_{\mathbf{K}} = \sum_{n,n',X,X',\nu,\nu'} h(n, X, \nu|n', X', \nu')\; a^{\dagger}_{n,X,\nu}\; a_{n',X',\nu'} \tag{14}$$

where,

$$\begin{aligned} h(n, X, \nu|n', X', \nu') = \;& \varepsilon_n \delta_{n,n'} \delta_{X,X'} \delta_{\nu,\nu'} + c_n c_{n'} \delta_{X,X'} \Big[- i\ \mathrm{sgn}(nn') \frac{\sqrt{2}}{l_B} \hbar v_4 \sqrt{|n'| - 1}\ \delta_{|n|,|n'|-1} \\ & - \mathrm{sgn}(n) \frac{\sqrt{2}}{l_B} \hbar v_3 \sqrt{|n'| + 1}\ \delta_{|n|,|n'|+2} - i\ \mathrm{sgn}(n') \gamma \delta_{|n|,|n'|-1} \\ & - i \frac{\sqrt{2}}{l_B} \hbar v_4 \sqrt{|n'|} \delta_{|n|,|n'|-1} \Big] \delta_{\nu,1}\ \delta_{\nu',2} + \delta_{\nu,2}\ \delta_{\nu',3} \Big[\delta_{X,X'-k_\theta l_B^2}\ A_{n,n'}\ e^{-iG'_{0,x}(X+X')/2} \\ & + \delta_{X,X'+k_\theta l_B^2/2}\ B_{n,n'}\ e^{-iG'_{1,x}(X+X')/2} + \delta_{X,X'+k_\theta l_B^2/2}\ D_{n,n'}\ e^{-iG'_{2,x}(X+X')/2} \Big] + \text{h.c.}. \end{aligned} \tag{15}$$

Similarly, we can find the matrix Hamiltonian for $\mathbf{K'}$ valley.

Notice that the Hamiltonian in Eq. (14) can be made to be periodic,i.e.,

$$h(n, X, \nu|n', X', \nu') = h(n, X + \Delta X, \nu|n', X' + \Delta X, \nu') \tag{16}$$

if $\Delta X = q k_\theta l_B^2/2$ where q is an integer. The above condition of discrete translational symmetry of the Hamiltonian can also be rewritten as,

$$\frac{q}{p} = \frac{6\phi}{\phi_0} \tag{17}$$

where p is an integer mutually coprime to q and ϕ_0 is the flux quantum. This equation is basically the condition that a moire unit cell contains a rational fraction of the flux quantum. The size of the matrix that we need to diagonalize is $4 \times q \times N_{LL}$, N_{LL} is the number of Landau levels of monolayer graphene. One requires a cutoff for the number of Landau levels in performing the numerical calculation. We choose this cutoff such that the energy spectrum and the gaps found in the low energy sector does not change with further increase of Landau levels. As we go to lower magnetic flux per unit cell, we require larger cutoff. The minimum number of Landau levels we have used is 31 when the magnetic flux per unit cell is close to 1. We have used 151 Landau levels when the magnetic flux ϕ/ϕ_0 is lower that 0.1.

As discussed in the main manuscript, our theoretical calculations capture the experimental results very well. While we see some small quantitative disagreement, we attribute that to the extra potential on each layer in a realistic sample induced by the presence of other layers. As a result, the effective potential on each layer could be different from the applied potential. In principle, a self-consistent calculation could capture the actual potential present in the layers. While such computation is numerically quite expensive, the resulting solutions will simply differ by certain shifts of potential.

Supplementary Note 3: Extraction of Hofstadter gaps from experimental data

To estimate the Hofstadter gaps we measure the variation of σ_{xx} as a function of ν for different temperatures at a constant magnetic field as shown in Supplementary Fig. 2a. Then we identified the σ_{xx} minima corresponding to the Chern gaps and plot the σ_{xx} magnitude as a function of temperature (T) for different Chern gaps as shown in Supplementary Fig. 2b. Now by fitting Arrhenius activation formula $\sigma_{xx} \propto \exp\{-\Delta_g/(2k_{\mathrm{B}}T)\}$ to the linear region in σ_{xx} vs $1/T$ curve we extract the gap Δ_g of the corresponding Chern gaps (see Supplementary Fig. 2c). Here, k_{B} is the Boltzmann constant. We repeat the analysis for different values of D and summarize the extracted gap at different (ν, D) points in Supplementary Fig. 2d.

Supplementary Note 4: Additional magneto-transport data

In Supplementary Fig. 3, we show the color-scale plots of σ_{xy} as a function of ν and D for three different values of B. The corresponding color-scale plots of σ_{xx} are shown in Fig. 1 in the main text. In Supplementary Fig. 4, we have marked additional Chern gaps with values of (C, s) corresponding to the same data used in Fig. 2a in the main text. Here, C is the Chern number and s is an integer denoting the moiré filling factor corresponding to the number of carriers per moiré unit cell in zero magnetic field.

Supplementary Fig. 5b and 5c show ν lineslices of the σ_{xx} and the corresponding σ_{xy} across the $s = -2$ and $s = 0$ Chern gaps at fixed magnetic fields. A dip in σ_{xx} in the blue-shaded region shows a (C, s) Chern gap. Measured values of σ_{xy} is close to Ce^2/h for each of the (C, s) Chern states showing approximate quantization. As mentioned in the main text, we do not see a clear quantization in σ_{xy} for all (C, s) states possibly due to small values of Hofstadter gaps and twist angle-inhomogeneity disorder. In Supplementary Note 6, we have discussed the role of flat bands in setting small Hofstadter gaps.

We observe electric field tunable Chern gaps across a wide range of twist angles $(1.09° - 1.46°)$ in multiple devices. In the main text and in Supplementary Figs. 3-6, we have used a device with twist angle 1.10° (device 1). In Supplementary Fig. 6, we show the details of fittings to extract the (C, s) states. In Supplementary Fig. 7, we show the evolution of Chern gaps from another device (device 2) with a twist angle of 1.09°. Though some of the Chern gaps are not fully developed possibly due to twist-angle disorder, the evolution of Chern gaps is overall similar to the data from device 1 in Supplementary Fig. 4. Supplementary Fig. 8 shows data from device 3 with a twist angle of 1.46°. In this device, we clearly see a correlated gap at $\nu = 2$ at zero magnetic field, as seen in Supplementary Fig. 8b. From Supplementary Fig. 8c-d, we see Chern gaps that tunes with the electric field. Observation of tunable Chern gaps is further repeated for another twist angle of 1.42° in Supplementary Fig. 9.

Supplementary Note 5: Evolution of the CNP gap and the role of tunable layer polarization

As discussed in the main text, the electric field tunable layer polarization plays an important role in TDBG. In particular, the tunable layer polarization can lead to multiple closings and reopening of a gap, as the energy levels disperse nonmonotonically with the electric field. To demonstrate this, in Supplementary Fig. 10a we show a color-scale plot of σ_{xx} at the CNP as a function of D and B from device 1. Few line slices of σ_{xx} vs. D at fixed values of B are shown in Supplementary Fig. 10b. For $B = 0$ T we note σ_{xx} remains high for $|D|/\epsilon_0 \lesssim 0.23$ V/nm suggesting that CNP gap opens up only after a finite electric field. However, at a moderate magnetic field above 2 T, σ_{xx} becomes small with a dip at $D = 0$, indicating the opening of the CNP gap even at zero electric field. At higher magnetic fields such as at $B = 9$ T, the CNP gap closes and reopens multiple times.

To examine the role of tunable layer polarization in TDBG we contrast with the case of BLG[12,13], where the occupied lowest LL's for $\nu = 0$ undergo a simple evolution over most of the range of D. As shown in the schematic of Supplementary Fig. 10c, electrons from $K(K')$ valleys occupy upper (lower) layer. At a finite B and low electric field (region I) the occupied states support spins in the two valleys which are antiferromagnetically correlated but cant into the magnetic field direction. However, as the electric field is increased, the energy of the LLs from opposite valleys disperse in opposite directions monotonically due to the layer-valley locking. This monotonic dispersion results in a near gap-closing at D^*, around which there may be other gapped or gapless phases in a narrow range of D due to interactions[14–16]. Outside this transition region a layer-polarized phase quickly emerges at higher electric fields (region II). The evolution of σ_{xx} in the parameter space of B and D for BLG is schematically shown in Supplementary Fig. 10d; the CNP gap in TBG shows similar evolution[17].

Now we turn to the case of TDBG which can be considered as two copies of BLG. In contrast to BLG, as shown in Supplementary Fig. 10e, a strong hybridization between the layers due to twisting ensures that the LLs from the two valleys do not fully layer-polarize in the same range of D as for BLG. The polarization varies as the electric field changes the hybridization, and consequently the nonmonotonic evolution of the LLs results in a complex evolution of the CNP gap as shown in Supplementary Fig. 10a and schematically in Supplementary Fig. 10f.

We further note the unique evolution of the CNP gap with B. Unlike BLG, where CNP gap is enhanced with B, TDBG shows a different trend. As seen in Supplementary Fig. 10g, σ_{xx} first decreases indicating the enhancement of the CNP gap with B. However, at high B, σ_{xx} increases indicating a gap closing. This is consistent with the Hofstadter energy spectrum for a topological band, where the gap with a nonzero Chern number C closes at $\Phi/\Phi_0 \leq 1/|C|$[18].

Supplementary Note 6: Role of flat band energy scale on resolving Hofstadter spectra

The narrow bandwidth of the flat bands sets a small energy scale in TDBG, resulting in small values of the Hofstadter gaps. To understand the effect of flat band, we plot two fan diagrams for two different values of D from device 3 with a twist angle of 1.46° in Supplementary Fig. 11. We find that the fan diagram for higher magnitude of electric field has more number of resolved Hofstadter gaps, consistent with the fact that the bandwidth of the flat bands increases with the electric field magnitude in TDBG[19]. Furthermore, we observe more number of Hofstadter gaps resolved on the hole side of the fan diagram, as seen in Supplementary Fig. 11b. This is because the bandwidth of the valence flat band is higher than that of the conduction flat band, as reflected in the observation of $\nu = 2$ correlated gap only on the electron side (see Supplementary Fig. 8).

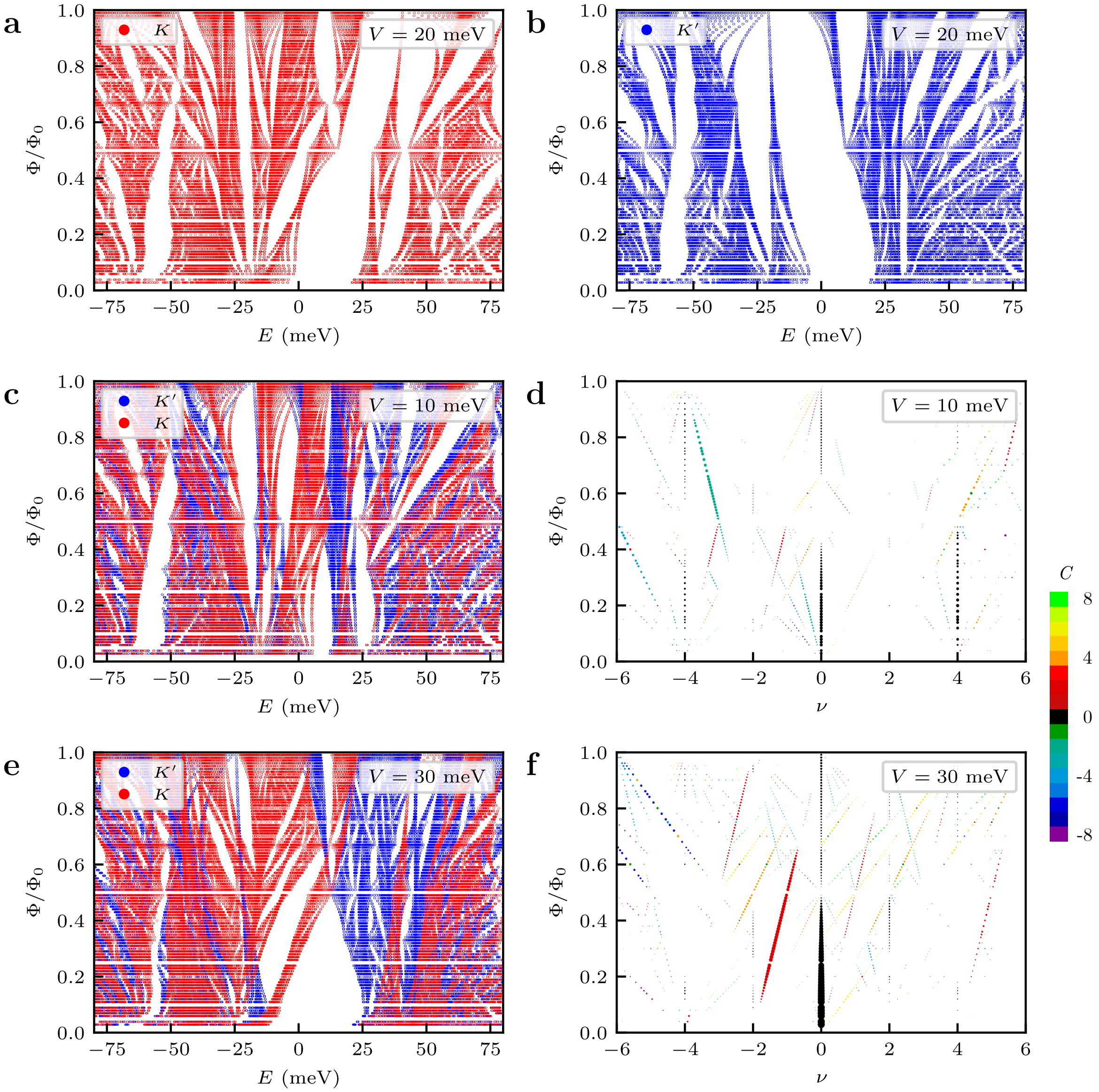

Supplementary Figure 1: Calculated Hofstadter spectra and Wannier diagrams for TDBG with twist angle 1.10° a,b, Hofstadter spectra of K and K' valleys, respectively, for an interlayer potential of $V = 20$ meV. The spectra combined from both valleys is shown in Fig. 3a in the main text with corresponding Wannier diagram in Fig. 3b. **c,e,** Hofstadter spectra considering both K and K' valleys for $V = 10$ meV and 30 meV, respectively. **d,f,** Wannier diagrams extracted from **c** and **e**.

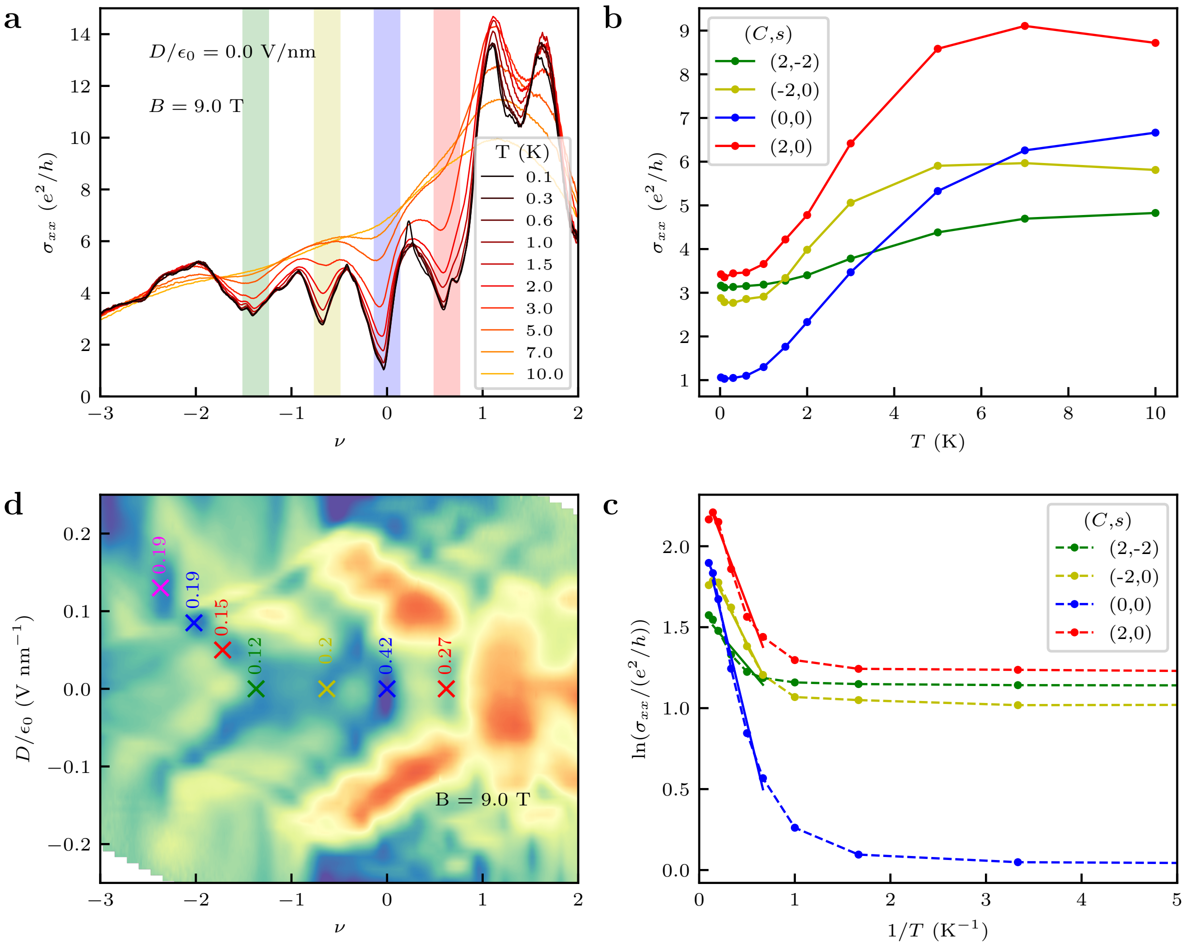

Supplementary Figure 2: Estimation of LL gaps. a, Variation of σ_{xx} vs. ν for different values of temperature at $B = 9$ T and $D/\epsilon_0 = 0$ V/nm. **b,** Temperature dependence of σ_{xx} minima for four different LL gaps at $B = 9$ T and $D/\epsilon_0 = 0$ V/nm extracted from **a**. **c,** Extraction of LL gap by fitting the linear region in $\ln \sigma_{xx}$ vs. $1/T$ plots obtained from **b**. Solid lines represent the linear fit. **d,** Values of extracted LL gaps in meV at some points in ν-D parameter space at $B = 9$ T.

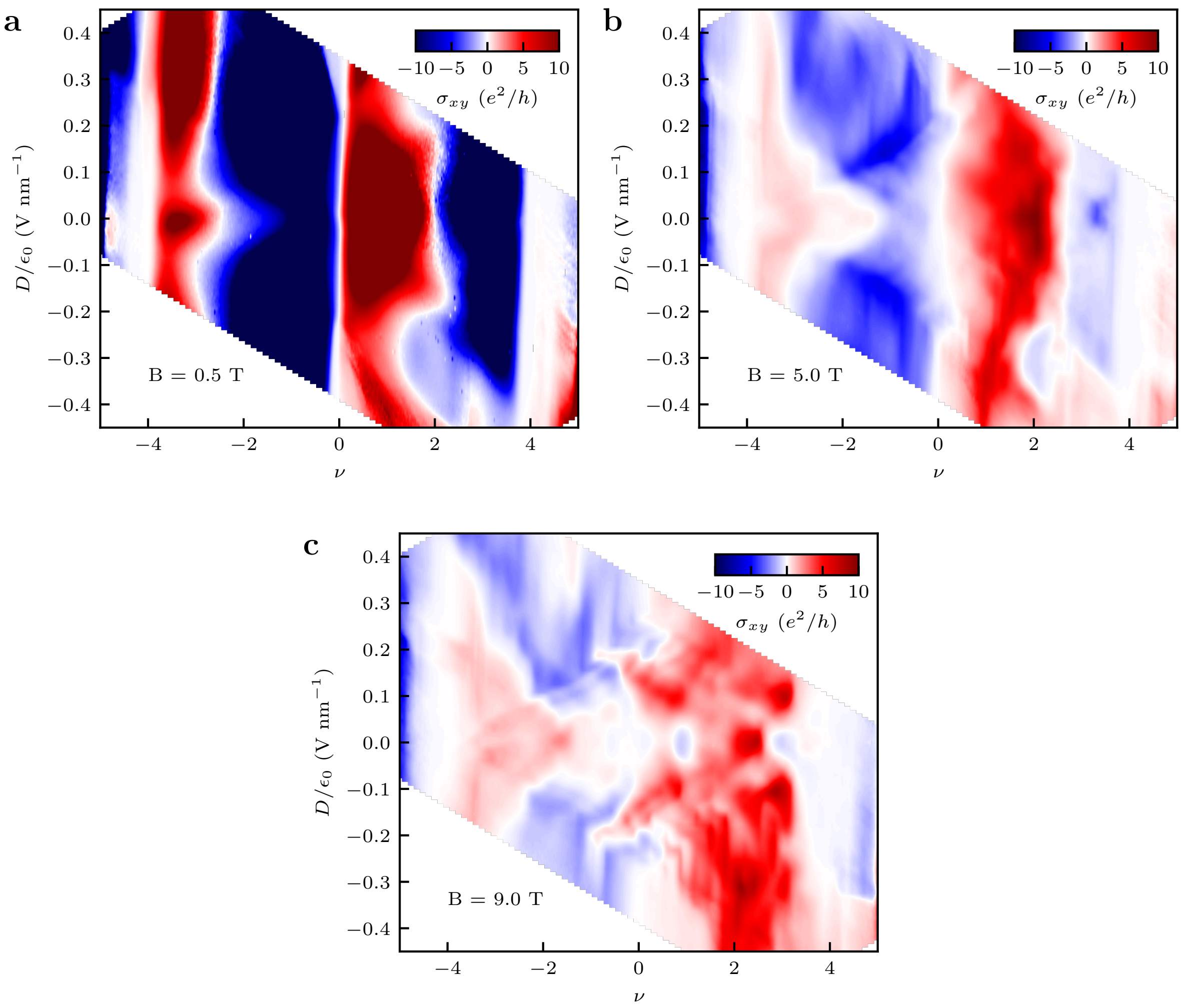

Supplementary Figure 3: Evolution of σ_{xy}. a,b,c, Color-scale plot of σ_{xy} vs. ν and D for three different values of magnetic field.

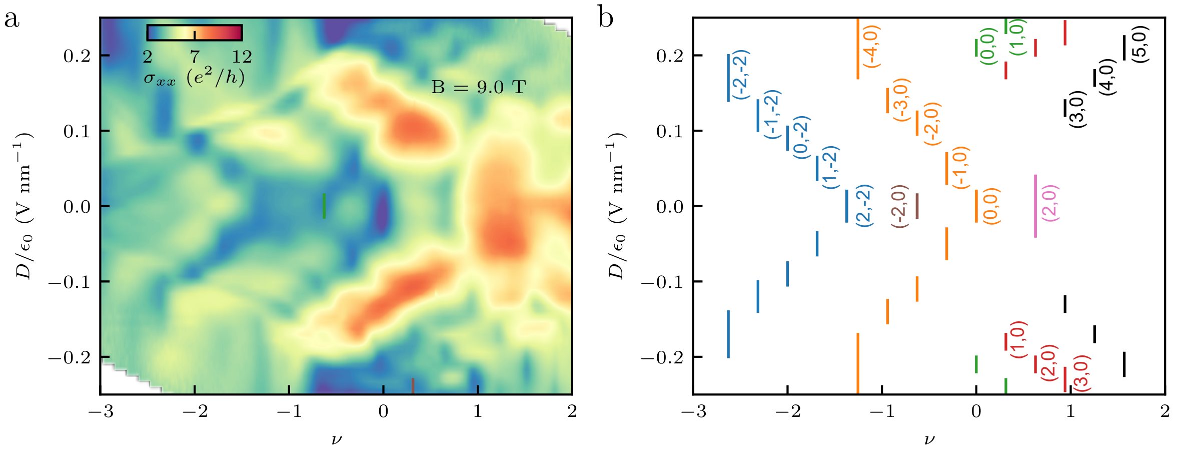

Supplementary Figure 4: Marking additional Chern gaps for device 1 with twist angle 1.10° used in the main text. a, Color-scale plot of σ_{xx} as a function D and ν at $B = 9$ T, as plotted in Fig. 2a of the main text, but without marking Chern gaps. **b,** The extracted values of (C, s) corresponding to σ_{xx} dips in **a**. Here, C is Chern number and s is an integer denoting the moiré filling factor corresponding to the number of carriers per moiré unit cell in zero magnetic field. The Chern gaps labeled with blue and orange colors are what we emphasized in the main text; here we have marked additional Chern gaps with different colors.

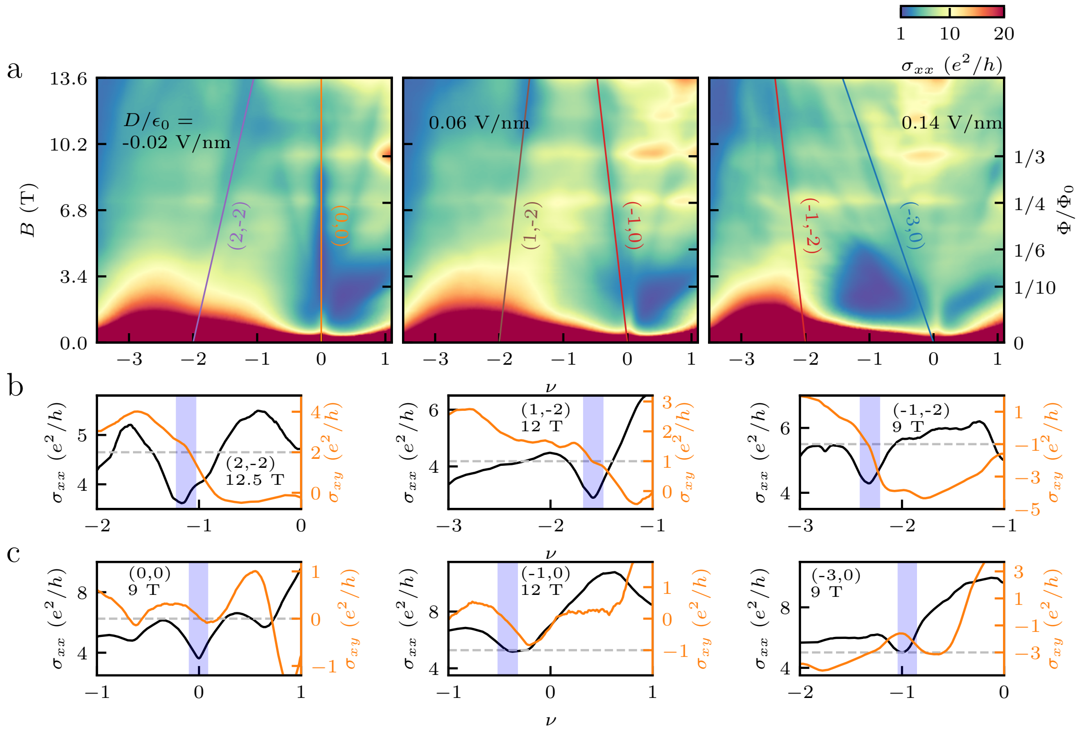

Supplementary Figure 5: Approximate quantization of (C, s) states. a, Fan diagrams reproduced from Fig. 2b of the main manuscript, with solid lines overlaid on σ_{xx} minimas, indicating the (C, s) Chern gaps. Line slices of σ_{xx} (black colored plot corresponding to the left axis) and σ_{xy} (orange colored plot corresponding to the right axis) vs. filling (ν) at fixed magnetic field for $s = -2$ (b) and $s = 0$ (c) Chern gaps. The dashed horizontal lines are a guide to the eye, corresponding to σ_{xy}= Ce^2/h. The blue-shaded ν window (with a width of 0.2) is centered at the ν value calculated from the Diophantine equation for a particular (C, s) state at that B value. σ_{xx} shows a dip within the blue ν-window due to each (C, s) Chern gap. The magnitude of measured σ_{xy} being close to the corresponding values of Ce^2/h in (b) and (c) shows approximate quantization.

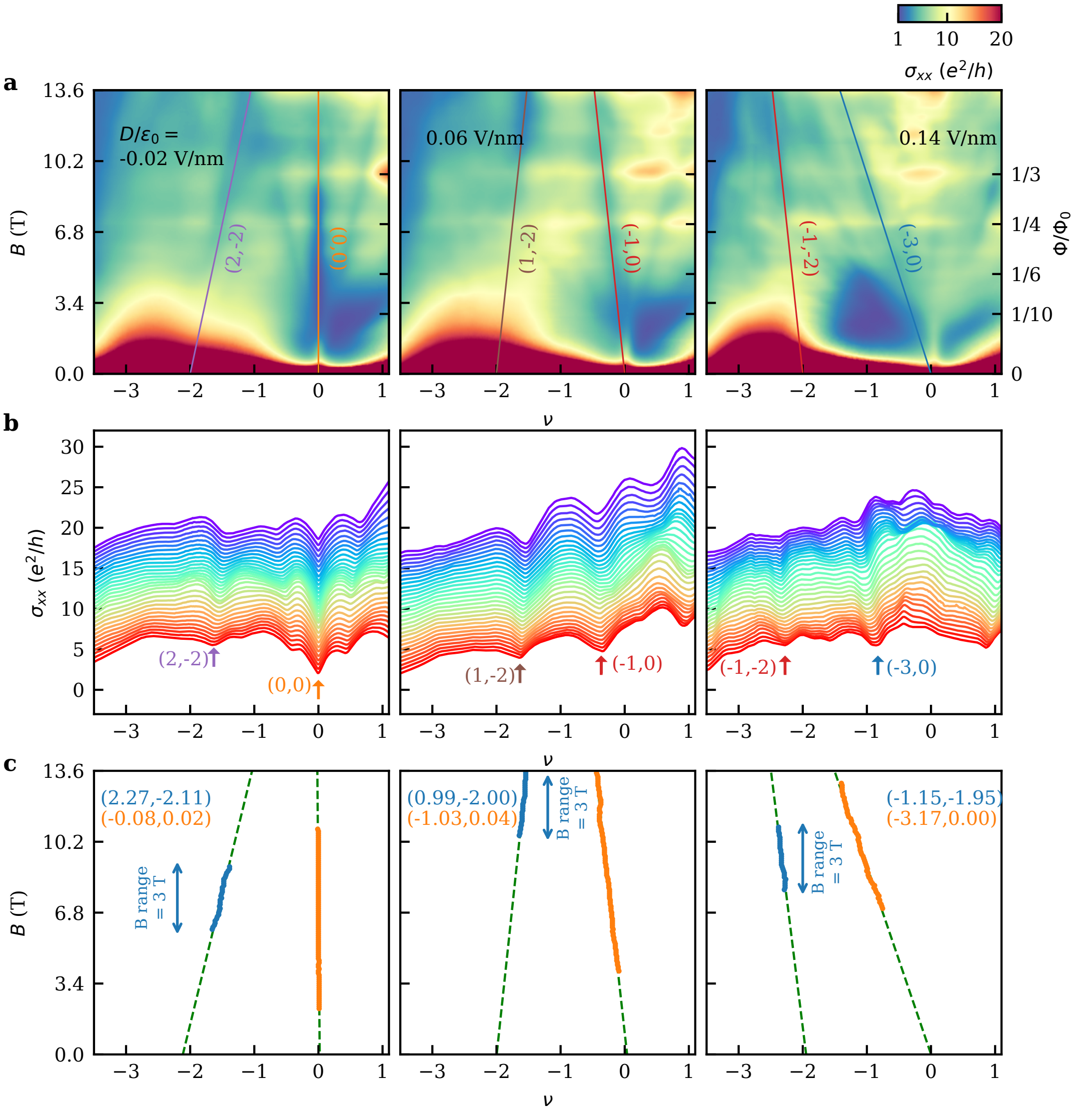

Supplementary Figure 6: Details of fitting to extract (C, s) for device 1 with twist angle 1.10° used in the main text. a, Fan diagrams reproduced from Fig. 2b of the main manuscript. **b,** σ_{xx} vs ν plots where magnetic field (B) is varied in steps of 0.1 T, showing clear minima in σ_{xx} corresponding to different (C, s) states as indicated by the arrows. The three different plots are for the three different electric fields used in **a**. Within a particular sub-panel, each σ_{xx} vs ν plot is shifted up by 0.5 units. **c,** Fitting of the extracted (ν, B) points of σ_{xx} minima for the different (C, s) states. The blue (orange) dots indicates σ_{xx} minima for $s = -2$ $(s = 0)$ state. The extracted (C, s) values are indicated in each plot. The corresponding σ_{xx} vs ν plots from which the σ_{xx} minima for $s = -2$ states are extracted are shown in **b**. Thirty-one σ_{xx} vs ν slices are used to fit each $(C, s = -2)$ state.

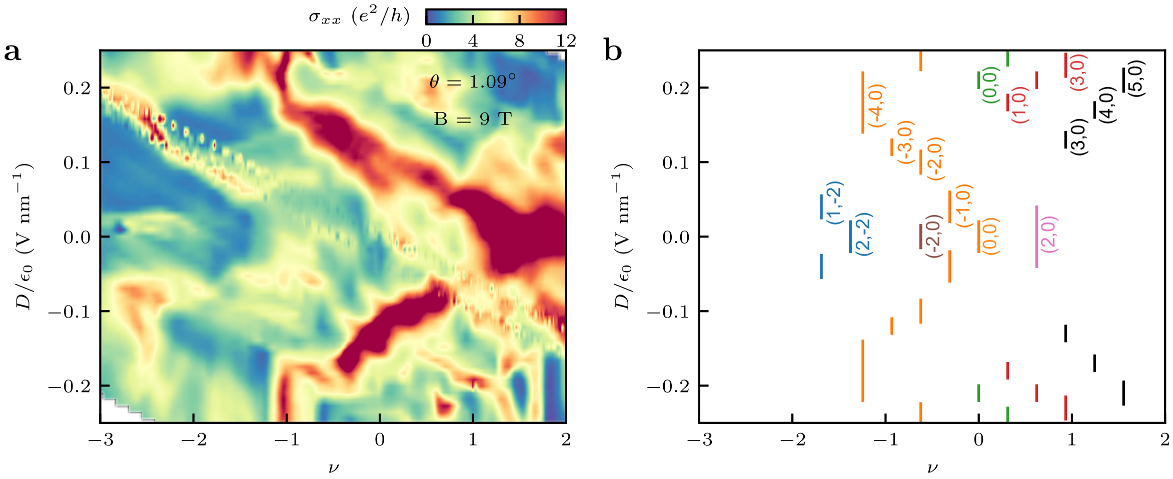

Supplementary Figure 7: Electric field tunable Chern gaps from device 2 with twist angle 1.09°. a, Evolution of σ_{xx} at 9 T showing multiple peaks/dips corresponding to Chern gaps evolving with the electric field. **b,** Values of (C, s) corresponding to the σ_{xx} dips in **a**. Overall, the evolution is similar to that for device 1 (Supplementary Fig. 4).

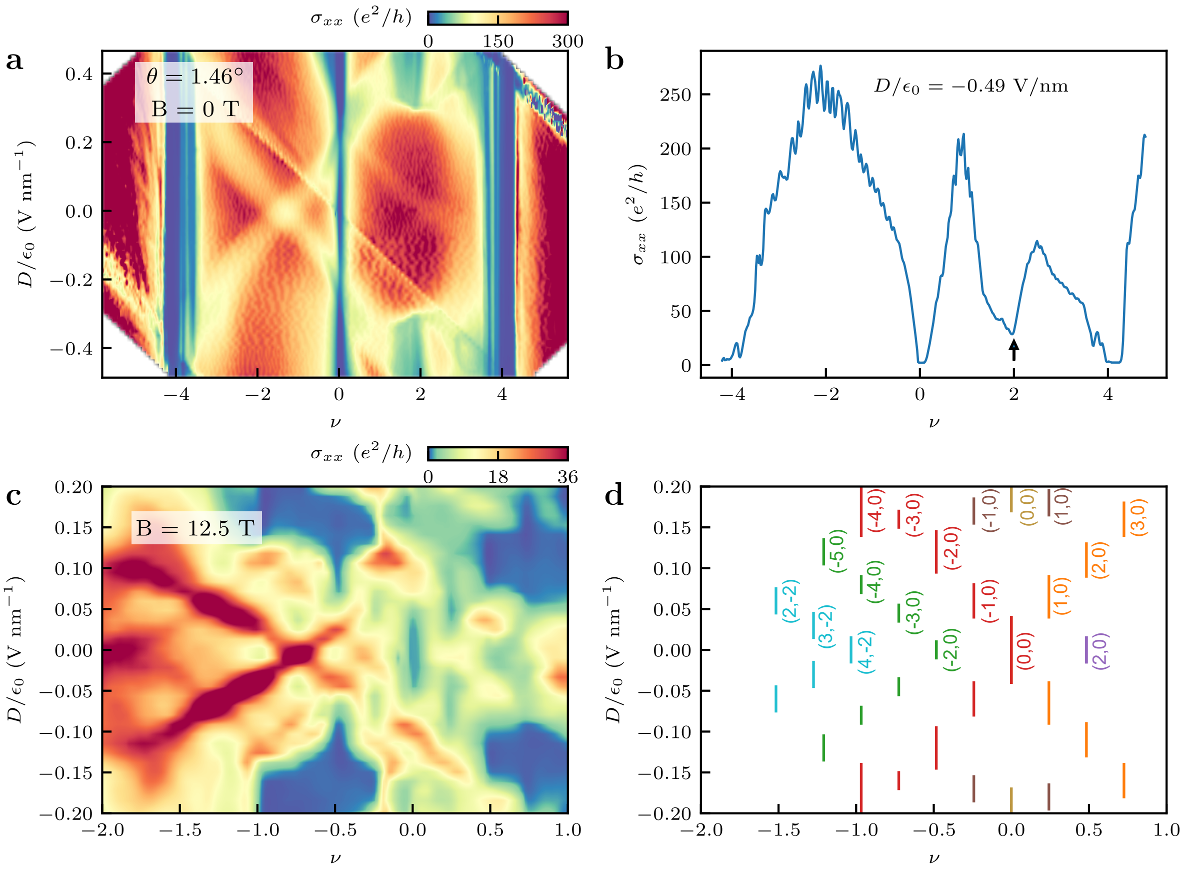

Supplementary Figure 8: Correlated insulator state and electric field tunable Chern gaps from device 3 with twist angle 1.46°. a, σ_{xx} as a function of ν and D at zero magnetic field. Correlated gap appears at $\nu = 2$ for $|D|/\epsilon_0 \sim 0.4$ V/nm. **b,** A line slice at $D/\epsilon_0 = -0.49$ V/nm clearly showing a dip in σ_{xx} at $\nu = 2$. **c,** Evolution of σ_{xx} at a finite magnetic field of 12.5 T showing multiple peaks/dips corresponding to Chern gaps evolving with the electric field. **d,** (C, s) values corresponding to the σ_{xx} dips in **c**.

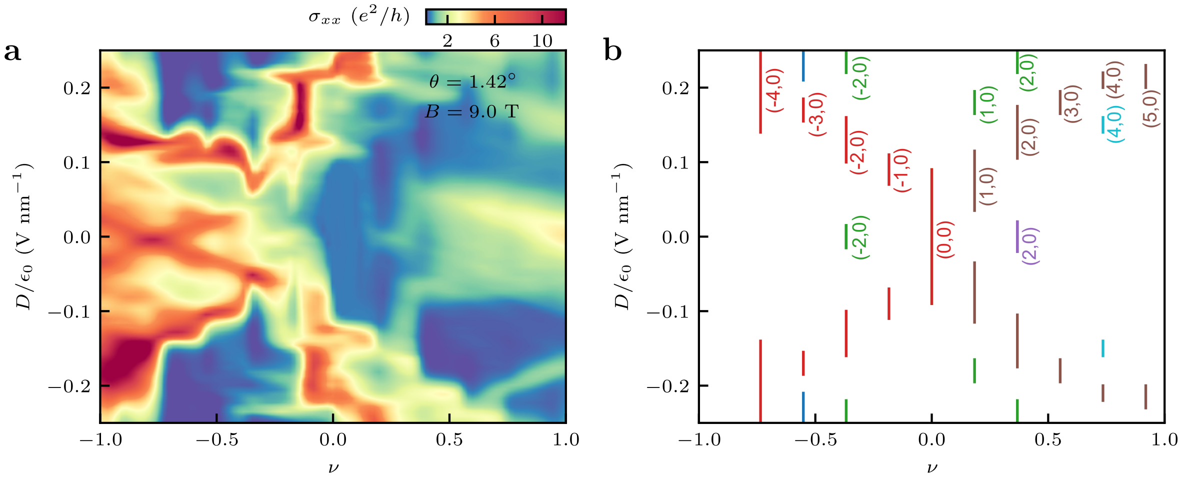

Supplementary Figure 9: Electric field tunable Chern gaps from device 3 with twist angle 1.42°. a, Evolution of σ_{xx} at 9 T showing multiple peaks/dips corresponding to Chern gaps evolving with the electric field. **b,** (C, s) values corresponding to the σ_{xx} dips in **a**.

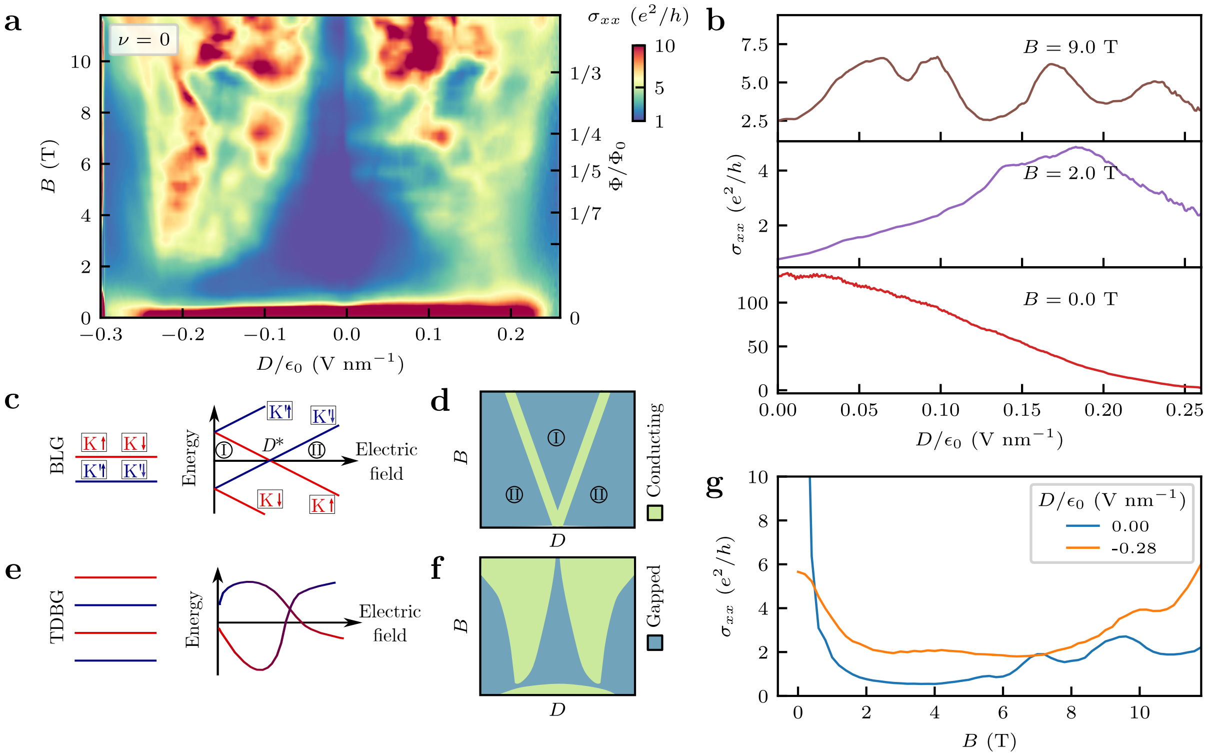

Supplementary Figure 10: Evolution of the CNP gap. a, Color-scale plot of σ_{xx} as a function D and B at $\nu = 0$. **b,** Line slices of σ_{xx} vs. D at $\nu = 0$ for few magnetic field values. **c,** Schematic showing the evolution of CNP gap in BLG from spin-polarized state (region I) to layer-polarized state (region II) as D is increased. The LLs from two valleys are locked to two layers and thus LL energy changes monotonically with D. **d,** Map of the gapped and conducting regions in the parameter space of B and D at CNP for BLG[12]. **e,** In TDBG two copies of BLG are hybridized. Thus LLs from different valleys are not locked to any particular layer – layer polarization is varied with D. Tunable polarization results in nonmonotonic evolution of the energy levels giving rise to multiple closing and reopening of the gap. **f,** A simplified schematic version of **a** to contrast the evolution of the CNP gap with that in BLG. **g,** Line slices from **a** showing σ_{xx} vs. B at $\nu = 0$ for two electric field values. The evolution of σ_{xx} indicates that the CNP gap first increases and then decreases as B is increased suggesting gap opening and closing as a function of B.

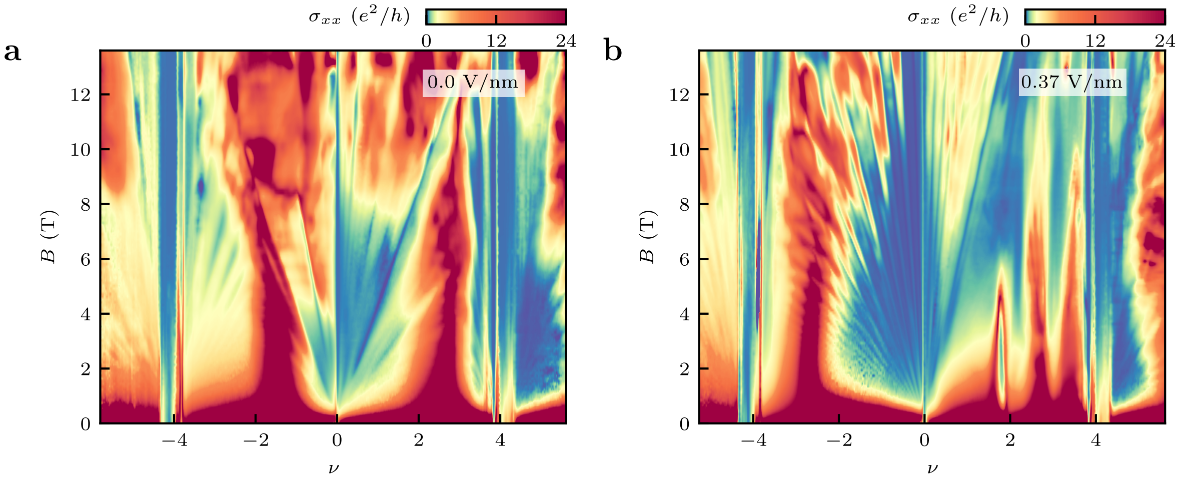

Supplementary Figure 11: Role of flat band energy scale on resolving Hofstadter spectra. a-b, Fan diagrams from device 3 with twist angle 1.46° for two different values of D: 0.0 V/nm (**a**) and 0.37 V/nm (**b**). More gaps are resolved for higher electric field due to higher bandwidth.

References

[1] Bistritzer, R. & MacDonald, A. H. Moiré bands in twisted double-layer graphene. *Proceedings of the National Academy of Sciences* **108**, 12233–12237 (2011).

[2] Lopes dos Santos, J. M. B., Peres, N. M. R. & Castro Neto, A. H. Graphene bilayer with a twist: Electronic structure. *Phys. Rev. Lett.* **99**, 256802 (2007).

[3] Lopes dos Santos, J. M. B., Peres, N. M. R. & Castro Neto, A. H. Continuum model of the twisted graphene bilayer. *Phys. Rev. B* **86**, 155449 (2012).

[4] Koshino, M. Band structure and topological properties of twisted double bilayer graphene. *Phys. Rev. B* **99**, 235406 (2019).

[5] Chebrolu, N. R., Chittari, B. L. & Jung, J. Flat bands in twisted double bilayer graphene. *Phys. Rev. B* **99**, 235417 (2019).

[6] Moon, P. & Koshino, M. Optical absorption in twisted bilayer graphene. *Phys. Rev. B* **87**, 205404 (2013).

[7] Koshino, M. & Moon, P. Electronic properties of incommensurate atomic layers. *Journal of the Physical Society of Japan* **84**, 121001 (2015).

[8] Moon, P. & Koshino, M. Energy spectrum and quantum hall effect in twisted bilayer graphene. *Phys. Rev. B* **85**, 195458 (2012).

[9] Crosse, J. A., Nakatsuji, N., Koshino, M. & Moon, P. Hofstadter butterfly and the quantum hall effect in twisted double bilayer graphene. *Phys. Rev. B* **102**, 035421 (2020).

[10] Bistritzer, R. & MacDonald, A. H. Moiré butterflies in twisted bilayer graphene. *Phys. Rev. B* **84**, 035440 (2011).

[11] Hejazi, K., Liu, C. & Balents, L. Landau levels in twisted bilayer graphene and semiclassical orbits. *Physical Review B* **100**, 035115 (2019).

[12] Weitz, R. T., Allen, M. T., Feldman, B. E., Martin, J. & Yacoby, A. Broken-Symmetry States in Doubly Gated Suspended Bilayer Graphene. *Science* **330**, 812–816 (2010).

[13] Kim, S., Lee, K. & Tutuc, E. Spin-polarized to valley-polarized transition in graphene bilayers at $\nu = 0$ in high magnetic fields. *Physical Review Letters* **107**, 016803 (2011).

[14] Hunt, B. M. *et al.* Direct measurement of discrete valley and orbital quantum numbers in bilayer graphene. *Nature Communications* **8**, 948 (2017).

[15] Murthy, G., Shimshoni, E. & Fertig, H. A. Spin-valley coherent phases of the $\nu = 0$ quantum hall state in bilayer graphene. *Phys. Rev. B* **96**, 245125 (2017).

[16] Li, J. *et al.* Metallic phase and temperature dependence of the $\nu = 0$ quantum hall state in bilayer graphene. *Phys. Rev. Lett.* **122**, 097701 (2019).

[17] Sanchez-Yamagishi, J. D. *et al.* Quantum Hall Effect, Screening, and Layer-Polarized Insulating States in Twisted Bilayer Graphene. *Physical Review Letters* **108**, 076601 (2012).

[18] Lian, B., Xie, F. & Bernevig, B. A. Landau level of fragile topology. *Physical Review B* **102**, 041402 (2020).

[19] Adak, P. C. *et al.* Tunable bandwidths and gaps in twisted double bilayer graphene on the verge of correlations. *Phys. Rev. B* **101**, 125428 (2020).
